# Influence of the Temperature and the Genotype of the *HSP90AA1* Gene over Sperm Chromatin Stability in Manchega Rams

**DOI:** 10.1371/journal.pone.0086107

**Published:** 2014-01-21

**Authors:** Manuel Ramón, Judit Salces-Ortiz, Carmen González, M. Dolores Pérez-Guzmán, J. Julián Garde, Olga García-Álvarez, Alejandro Maroto-Morales, Jorge H. Calvo, M. Magdalena Serrano

**Affiliations:** 1 CERSYRA, Valdepeñas. Spain; 2 INIA, Madrid, Spain; 3 Health and Biotechnology IREC (Consejo Superior de Investigaciones Científicas–University of Castile–La Mancha–Junta de Comunidades de Castilla-La Mancha), Albacete, Spain; 4 Unidad de Tecnología en Producción Animal, CITA, Zaragoza, Spain; University of Hawaii at Manoa, John A. Burns School of Medicine, United States of America

## Abstract

The present study addresses the effect of heat stress on males' reproduction ability. For that, we have evaluated the sperm DNA fragmentation (DFI) by SCSA of ejaculates incubated at 37°C during 0, 24 and 48 hours after its collection, as a way to mimic the temperature circumstances to which spermatozoa will be subject to in the ewe uterus. The effects of temperature and temperature-humidity index (THI) from day 60 prior collection to the date of semen collection on DFI were examined. To better understand the causes determining the sensitivity of spermatozoa to heat, this study was conducted in 60 males with alternative genotypes for the SNP G/C_−660_ of the *HSP90AA1* promoter, which encode for the Hsp90α protein. The Hsp90α protein predominates in the brain and testis, and its role in spermatogenesis has been described in several species. Ridge regression analyses showed that days 29 to 35 and 7 to 14 before sperm collection (bsc) were the most critical regarding the effect of heat stress over DFI values. Mixed model analyses revealed that DFI increases over a threshold of 30°C for maximum temperature and 22 for THI at days 29 to 35 and 7 to 14 bsc only in animals carrying the GG_−660_ genotype. The period 29–35 bsc coincide with the meiosis I process for which the effect of the Hsp90α has been described in mice. The period 7–14 bsc may correspond with later stages of the meiosis II and early stages of epididymal maturation in which the replacement of histones by protamines occurs. Because of GG_−660_ genotype has been associated to lower levels of *HSP90AA1* expression, suboptimal amounts of *HSP90AA1* mRNA in GG_−660_ animals under heat stress conditions make spermatozoa DNA more susceptible to be fragmented. Thus, selecting against the GG_−660_ genotype could decrease the DNA fragmentation and spermatozoa thermal susceptibility in the heat season, and its putative subsequent fertility gains.

## Introduction

Increasing concern over the implications of Climate Change in biodiversity is clear. Many efforts are now intended to better understand such implications, which are reflected by the large number of studies about this topic developed in the last decade [1], [2], [3]. It is now generally acknowledged that climate change has a wide-range of biological consequences, potentially leading to impacts on biodiversity. These biological effects are especially noticeable in areas with adverse environmental conditions, such as the arid regions of southern Europe, where temperature and humidity conditions are more extreme. In these areas an important farming activity takes place. Climate can affect in many ways animals' ability to survive and to produce. In this context, breeding for heat stress tolerance is of interest.

Among others, climate factors can have diverse and often strong effects on reproduction efficiency, with obvious consequences in animal's fitness (see [4] for references) which can result, ultimately, in high economic losses for breeders [5], [6]. Focusing on male reproduction, exposure to adverse conditions of high temperature and humidity may led to a reduction of the number of spermatozoa [7], [8] and also to an impairment of their functionality [8], [9], which will be accompanied by a transient period of partial or complete infertility. After heat stress, viability of the spermatozoa may not be compromised but some of them will appear with DNA damage. Thus, a reduction in DNA integrity has been described in rams [10], as well as alterations in DNA, RNA and protein synthesis, and abnormal chromatin packing in mice [8], [11], [12] under heat stress conditions. Two singular characteristics differentiate sperm from somatic cells: protamination and absence of DNA repair mechanisms. During spermiogenesis, protamines replace the majority of histones [13]. This dense compacting gives protection against exogenous assault to the sperm DNA [14]. DNA repair in sperm is terminated as transcription and translation stop at post-spermiogenesis, so these cells have no mechanism to repair the damage occurred during their transit through the epididymis and post-ejaculation [15]. Therefore, assessing levels of DNA fragmentation can be a useful tool for evaluating the effects of heat stress on sperm and its consequences on male fertility. Sperm DNA fragmentation is considered a non compensable trait which implies that the pregnancy ratio does not change when the number of sperm inseminated increases [16], [17]. The relationship between sperm DNA fragmentation index (DFI) and male fertility has been studied in humans [18], [19], [20], bulls [21] and boars [22]. Thresholds for sub fertility were much lower for boars (6%) and bulls (14.2%) than that for humans (30%). Recently, in rams Nordstoga et al. [23] showed an association between sperm DNA integrity and the non returned rate in Norwegian cross-bred rams.

Sensitivity of mammalian germ cells to environmental heat stress has been extensively studied [24]. Sperm damage is influenced by the stage at which germ cells are exposed to stress [25]. Several authors have examined the sensitivity of testicular cell population to heat [12], [26], [27]. From histological studies it has been concluded that pachytene spermatocytes and early spermatids are the cells in the testis which are most susceptible to heat [28]. At the molecular level, there is some literature regarding sperm damage by heat in mice [29], [30], rats [31], [32] and monkeys [33]. Heat shock proteins are a family of highly conserved proteins that play a fundamental role in the maintenance of cellular homeostasis, under both physiological and stress conditions [34]. Heat shock proteins Hsp105, Hsp90, Hsp60 and Hsp27 have been linked with apoptosis and heat stress response processes in Sertoli cells, spermatogonias, spermatocytes and spermatids. Hsp90 (90 kDa heat shock protein) is an ubiquitous highly conserved protein comprising up to 2% of total cell proteins even under non-stressed conditions. In eukaryotes there are two cytosolic Hsp90 isoforms encoded by two separate genes, the Hsp90α (*HSP90AA1* gene) and the Hsp90β (*HSP90AB1* gene). Whereas Hsp90β is more or less constitutively and ubiquitously expressed, the expression of hsp90α is heat-inducible and more tissue specific [35]. Hsp90α predominates in the brain and testis, while hsp90β is enriched in other peripheral organs [36].

A role for Hsp90 in spermatogenesis was first described in *Drosophila melanogaster*, were males with certain transheterozygous combinations of mutant Hsp90 alleles are sterile and display a disrupted meiosis [37]. In mice, a requirement of the Hsp90α for spermatogenesis has been shown [38]. Authors pointed out that Hsp90α must be necessary at least during the first wave of spermatogenesis. In the absence of Hsp90α meiosis arrests very specifically towards the end of the pachytene stage, disassembling of homologous chromosomes fail and normal diplotene spermatocytes are totally absent. Also, an absence of a comparable phenotype in Hsp90α mutant females was observed [38]. Also in mice [39] a chaperoning function of the Hsp90 protein to assess the proper folding of tNASP (testis histone binding protein) to bind linker histones, have been observed. Expression of Hsp90 and tNASP precede the expression of H1t (histone subtype 1 restricted to the testis) in pachytene spermatocytes [40], [41], [42]. Authors [39] pointed out that after the synthesis of linker histones in the cytoplasm they are bound to a complex containing NASP and Hsp90. NASP-H1 is subsequently released from the complex and translocated to the nucleus where the H1 is released for bind DNA [43].

The gene (*HSP90AA1*) encoding the inducible form of the Hsp90α, was sequenced, mapped and characterized in sheep by Marcos-Carcavilla et al. [44]. Fifteen polymorphisms located at the gene promoter were detected [44], [45]. The transversion G/C located at position −660 in the gene promoter was associated with resistance/susceptibility to *scrapie* [46] and with the adaptation of several sheep breeds to the different thermal conditions in where they are reared [47]. In a recent work of this same group [48] several SNPs located at the *HSP90AA1* gene promoter were associated with differences in the expression rate of this gene in blood under mild and heat stress temperatures in rams. The CC-660 genotype was associated to the highest levels of *HSP90AA1* expression under heat stress conditions.

Therefore, the aim of the present study was to examine: 1) if heat stress has an effect on chromatin stability of ram's sperm, and; 2) if a differential response to heat stress occurs based on male's genotype for one polymorphism located at the *HSP90AA1* gene promoter. For that, semen samples from males with different genotypes for the *HSP90AA1* gene were collected and exposed to heat during 48 h. Daily temperature and relative humidity for the 60 days prior to semen collection were recorded and their effect on resistance/susceptibility of spermatozoa to heat stress were assessed. Finally, it was examined whether there was a sperm differential response to heat stress depending on the *HSP90AA1* genotype of males.

## Results

### Weather data

**Figure 1:**
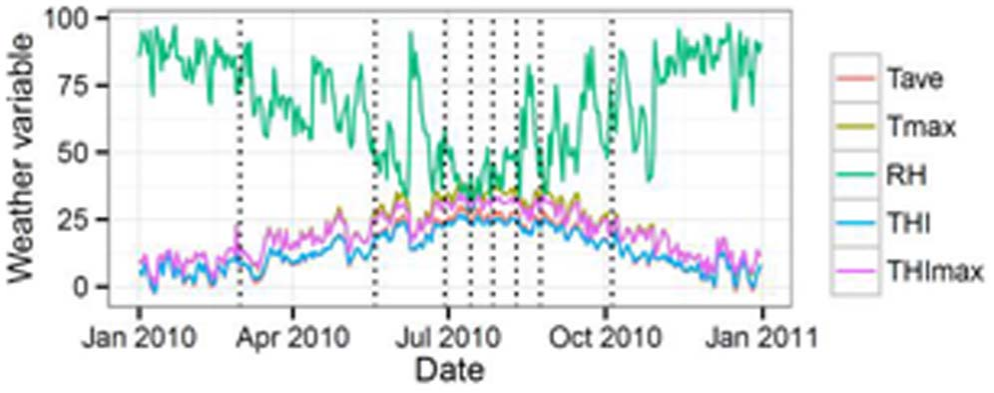
Trends of daily average (Tave, °C) and maximun (Tmax, °C) temperatures, relative humidity (RH, %) and average (THI) and maximum (THImax) temperature humidity index along the year 2010 (Data from SIAR http://crea.uclm.es/siar/datmeteo/). Dotted lines are days of semen collection.

Figure 1 shows the evolution of average (Tave) and maximum (Tmax) daily temperatures, average daily relative humidity (RH) and average of the Temperature Humidity Index (THI) along the period when sperm samples were collected. Average daily temperatures higher than 25°C and maximum daily temperatures higher than 30°C were observed from June to August. These temperatures exceed the 22.2°C temperature threshold over which heat stress is considered [49]. For the same period, minimum daily temperatures never dropped from 10°C. The highest values of RH were found from January to March (79 to 94%), however RH higher than 70% were observed at some points of the summer season (June, July and August), probably coinciding with summer storms. There were also maximum values of RH greater than 90% at June and August. From June to August THI, ranges from 22.4 to 27.0, which include the three THI heat stress categories, moderate (22.2 to 23.3), severe (23.3 to 25.6) and extreme (25.6 and over) [49]. If maximum daily THI is considered, we found days from May to August in which this parameter was in the range of extreme heat stress.

### DFI values

**Figure 2:**
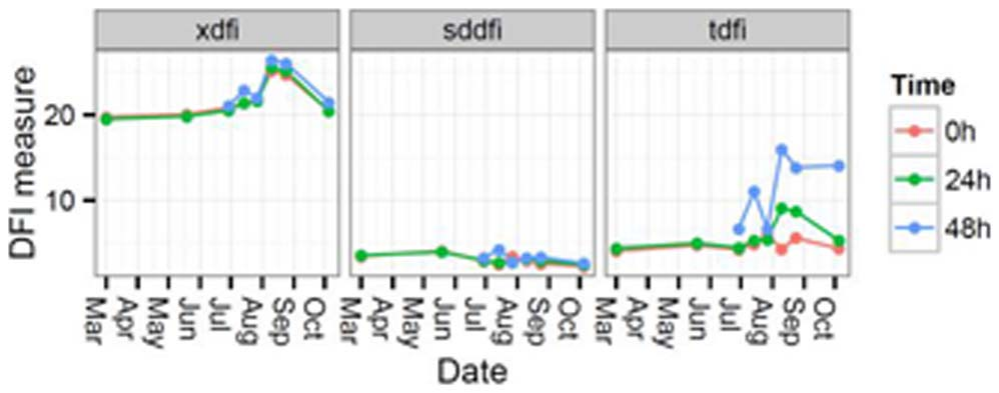
Changes in xDFI, sdDFI and tDFI values with the incubation time (0 h, 24 h and 48 h) along the period of the year from which sperm samples were collected.

Figure 2 shows the evolution of xDFI, sdDFI and tDFI values with the incubation time (0 h, 24 h and 48 h) along the period of the year from which sperm samples were collected. There were measures at 48 h of incubation time only for sperm samples collected from the end of May to October. The xDFI values for the three incubation times didn't show significant changes in sperm samples collected between March and the beginning of August (xDFI around 20–21). However, their values increased to 26 in August, dropping again to values of 20–21 in October. The sdDFI values did not experiment significant changes along the year for any incubation time, showing a very stable value close to 2.6. However, for tDFI values, differences along the year among alternative incubation times were observed. Thus, tDFI values did not show important changes along the period studied (1.9) for 0 h of incubation time (after semen collection), except a certain decrease observed in July (0.3). After 24 h of incubation, an average value of tDFI of 2.3 was found for samples collected from March to June. tDFI value decreased to 0.7 in July, and increased to 6.8 in August, dropping to 3.0 in October. Finally, for 48 h of incubation, an increase of tDFI from 4.4 to 6.4 was found for measures taken in June and July, and decreased to 2.1 for samples collected at the end of July. A clear increase of tDFI values was observed for samples collected in August, 13.7. In samples collected in October tDFI values dropped to 11.5. Thus, incubation times of 24 and 48 h lead to same trend on tDFI values but with different magnitude, being greater when sperm samples were incubated during more time.

### Ridge regression analysis

**Figure 3:**
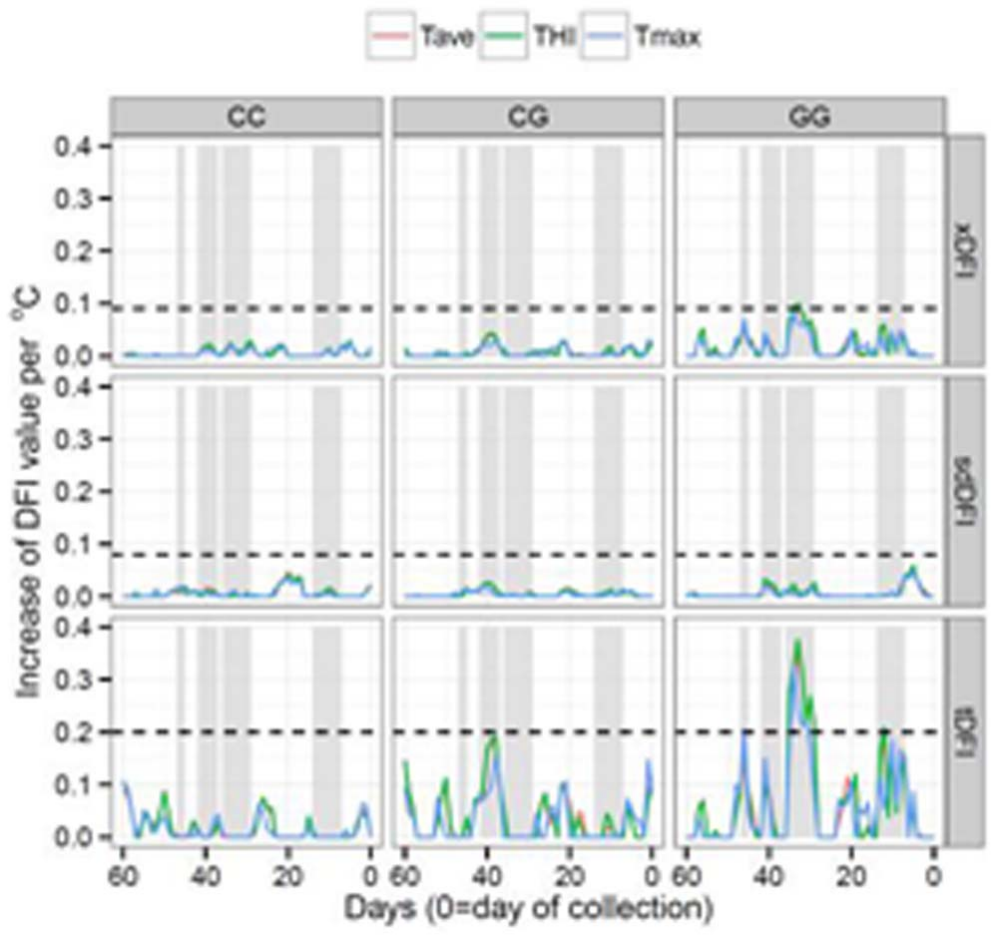
Ridge regression analyses relating DFI measures (xDFI, sdDFI and tDFI) from day 60 prior to semen collection to date of collection with weather measures (Tave  =  average daily temperature, Tmax = maximum daily temperature, and THI = temperature humidity index), for each *HSP90AA1* genotype. Fitted effects extending beyond dotted-lines (---) differ significantly (P<0.05) from zero. Four regions (gray regions) with a significant possible effect on sperm DFI levels were identified.

Figure 3 shows results from ridge regression analyses relating DFI measures to weather parameters for each *HSP90AA1* genotype. Results reveal a large effect on tDFI levels, a moderate to low effect on xDFI levels and no effect on sdDFI values. Regarding to *HSP90AA1* genotypes, CC_−660_ males did not show any significant change in DFI values associated with an increase of Temperature/THI within the 60 days prior to semen collection; for CG_−660_ males, a moderate effect was observed for tDFI levels; and finally, for GG_−660_ males, a clear effect of Temperature/THI was observed in the 60 days considered in this study. The period of time with the most influence over xDFI and tDFI values was that one located between days 29 to 35 before sperm collection (bsc), with the largest effect on day 33, for the three different weather parameters studied (Tave, Tmax and THI). For this period of time in GG_−660_ males, the estimated increase of DFI per °C or THI unit was 0.10 and 0.35 for xDFI and tDFI respectively. In addition to these days, other periods in which DFI levels underwent changes were identified. Thus, the time periods between days 7–14, 37–42 and 45–47 were also considered in this study, although none of them showed a clearly significant effect on the levels of DNA fragmentation of spermatozoa. An increase of tDFI levels close to 0.19 per °C or THI unit was estimated for the period 7 to 14 days bsc in GG_−660_ males; while an increase of tDFI levels of 0.18 was estimated for the days 37 to 42 in CG_−660_ males.

### Mixed model estimates

**Figure 4:**
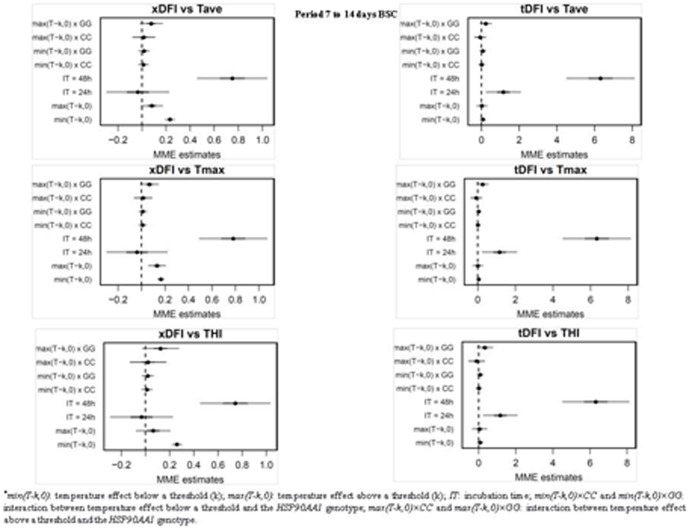
Regression coefficients from the mixed-effects model relating DFI values with summary measure of Tave, Tmax and THI for the days 7 to 14 before semen collection. For each coefficient in the model, estimates (points) plus and minus 1 (bold line) and 2 (thin line) standard deviations are represented. ^*^

**Figure 5:**
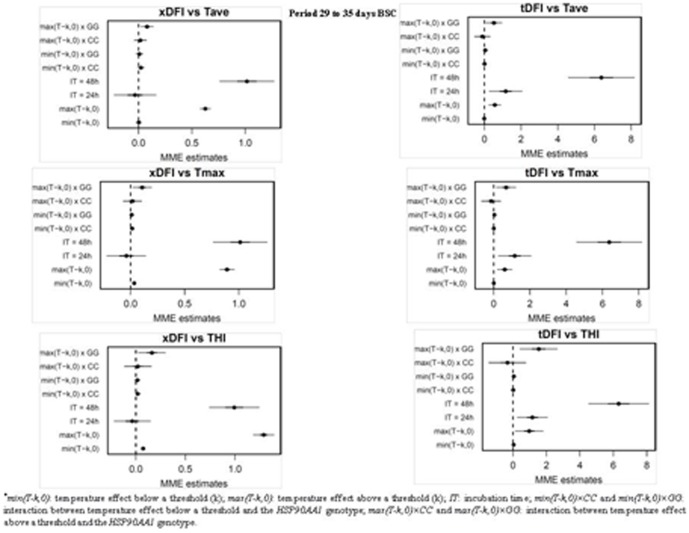
Regression coefficients from the mixed-effects model relating DFI values with summary measure of Tave, Tmax and THI for the days 29 to 35 before semen collection. For each coefficient in the model, estimates (points) plus and minus 1 (bold line) and 2 (thin line) standard deviations are represented. ^*^

Among the four time periods considered, the period between days 29 to 35 bsc showed the largest effect on the levels of sperm DNA fragmentation. Figures 4 and 5 show mixed-model estimates for the relationships between xDFI and tDFI sperm levels with temperature/THI for the days 7 to 14 and 29 to 35 bsc and *HSP90AA1* genotypes (numerical values are provided in Tables S1, S2, S3 and S4). Results for sdDFI measures were not presented as no significant changes associated with weather parameters were observed (Figure 3). Thresholds at which degree of DNA fragmentation significantly increased were estimated (median values for the periods considered; see Tables S1, S2, S3 and S4) at 22.6°C for Tave, 25.1 for THI and 29.9°C for Tmax when xDFI measured was considered, and at 21.8°C, 21.3 and 29.2°C for Tave, THI and Tmax, respectively, when evaluating the tDFI levels. The effects sperm incubation time at 48 h, temperature/THI over a threshold and the interaction of this temperature/THI with the GG_−660_ genotype for the *HSP90AA1* gene were significant (p<0.05) in all models evaluated. All males, independently of their genotype, showed a significant increase on DNA fragmentation when Temperature/THI exceeds the thresholds. However, for the interaction temperature/THI x genotype, only GG_−660_ males showed a significantly increase in DFI values (Table 1). These increments were around 0.1 and up to 0.4 per unit of Temperature/THI for the xDFI and tDFI levels, respectively, in the period from 7 to 14 days bsc, and around 0.2 and up to 1.3 per unit of Temperature/THI for the xDFI and tDFI levels, respectively, in the period from 29 to 35 days bsc. For the other two periods of time considered (37–42 and 45–47 days bsc), sperm DFI levels also increased with the incubation time (24 and 48 h) and at temperatures above a threshold (Figure S1), although the observed response for the three *HSP90AA1* genotypes was quite similar and of smaller magnitude than for the periods above mentioned (Table 1).

**Table 1:** Differences for the effect of the Temperature/THI on sperm DFI values between *HSP90AA1* genotypes.

			7 to 14	days		29 to 35	days
		CG - CC	GG - CC	GG - CG	CG - CC	GG - CC	GG - CG
Tave	xDFI	−0.02 _[0.750]_	0.06 _[0.190]_	0.08 _[0.110]_	−0.02 _[0.578]_	**0.07 _[0.032]_**	**0.09 _[0.007]_**
	tDFI	0.07 _[0.634]_	**0.30 _[0.050]_**	0.23 _[0.141]_	0.16 _[0.593]_	**0.95 _[0.002]_**	**0.79 _[0.011]_**
Tmax	xDFI	−0.01 _[0.741]_	0.04 _[0.177]_	0.05 _[0.095]_	−0.02 _[0.681]_	**0.09 _[0.039]_**	**0.11 _[0.014]_**
	tDFI	0.08 _[0.618]_	**0.34 _[0.029]_**	0.26 _[0.091]_	0.14 _[0.605]_	**0.82 _[0.003]_**	**0.68 _[0.013]_**
THI	xDFI	−0.02 _[0.770]_	0.10 _[0.180]_	0.12 _[0.110]_	−0.02 _[0.770]_	**0.14 _[0.045]_**	**0.16 _[0.022]_**
	tDFI	0.11 _[0.618]_	**0.44 _[0.048]_**	0.33 _[0.140]_	0.22 _[0.592]_	**1.30 _[0.002]_**	**1.07 _[0.011]_**

			37 to 42	days		45 to 47	days
		CG - CC	GG - CC	GG - CG	CG - CC	GG - CC	GG - CG
Tave	xDFI	−0.01 _[0.821]_	0.05 _[0.114]_	0.05 _[0.071]_	−0.01 _[0.800]_	0.05 _[0.390]_	0.06 _[0.270]_
	tDFI	0.11 _[0.610]_	0.14 _[0.530]_	0.03 _[0.900]_	0.03 _[0.810]_	0.13 _[0.300]_	0.10 _[0.430]_
Tmax	xDFI	0.00 _[0.530]_	0.05 _[0.670]_	0.06 _[0.076]_	−0.01 _[0.770]_	0.05 _[0.240]_	0.07 _[0.140]_
	tDFI	0.06 _[0.600]_	0.07 _[0.530]_	0.01 _[0.920]_	0.02 _[0.870]_	0.18 _[0.260]_	0.15 _[0.330]_
THI	xDFI	−0.04 _[0.744]_	0.20 _[0.108]_	0.24 _[0.054]_	−0.02 _[0.800]_	0.07 _[0.420]_	0.09 _[0.290]_
	tDFI	0.23 _[0.620]_	0.36 _[0.440]_	0.13 _[0.780]_	0.04 _[0.780]_	0.16 _[0.280]_	0.12 _[0.420]_

These differences correspond to the temperatures/THI above the threshold (*max(T-k, 0)*; see materials and methods). Mean differences and p-values (within brackets) are presented. Significant differences among genotypes are highlighted in bold.

## Discussion

The present study shows how the exposure of males to heat has consequences on the spermatogenesis process that result in an impairment of sperm chromatin which could lead to subfertile events. Exposure to temperatures above a threshold at certain stages of the spermatogenesis process leads to an increase of DNA fragmentation levels of spermatozoa. Moreover, here we show how the resistance/susceptibility of sperm chromatin to heat is subjected to the genotype of a SNP located at position −660 in the promoter region of the *HSP90AA1* gene. Thus, males with the GG_−660_ genotype have resulted to be more susceptible to environmental heat, showing significantly higher values of sperm DNA fragmentation.

In animals, where DNA damage can be experimentally induced in the paternal germ line, strong associations have been shown between the damage of the paternal genome and embryo development including effects on the new born and subsequent generations [50], [51]. Chromatin in rams was shown to be more decondensated in summer, but no differences were observed between breeding (September to January) and non breeding (February to June) seasons [52]. In addition, the considerable increase in DFI parameters in summer suggests that chromatin may be more susceptible to denaturation in this period.

Our results have showed moderate tDFI values at the beginning of June, decreasing at the end of July, with a clear increase on August and again a decrease in October for semen samples incubated during 24 and 48 h, being greater when sperm samples were incubated during more time. Levels of DNA fragmentation in sperms sample are consequence of outdoor temperatures occurred during the spermatogenesis (60 days prior to semen collection) and heat stress derived of the incubation at 37°C during 24 or 48 hours. The experimental design proposed in the present study was aimed to account for both sources of DNA fragmentation. We performed 7 semen collections from March to October, seeking days with different conditions of temperature (comfort/heat). On the one hand, climate information for the 60 days prior to semen collection was collected, in order to identify which days are more related to the degree of DNA fragmentation. On the other hand, sperm DNA fragmentation was assessed just after collection and after 24 and 48 hours of incubation in an environment inducing thermal stress (37°C) in order to evaluate the response of spermatozoa to heat stress under environmental conditions that mimic the temperature circumstances to which spermatozoa are subject to into the ewe uterus. It is described that an exposition to heat during spermatogenesis stimulates the synthesis of the Hsp90 protein in those males with CC-660 genotype, whereas basal levels are maintained in males with the GG-660 genotype. Therefore, it is expected that the latter would not be able to respond appropriately to heat stress during incubation (24–48 h at 37°C) increasing their levels of DNA fragmentation. Our experimental design allows relating these two measures: the in vitro heat stress and outdoor temperatures. Changes on tDFI levels derived from changes on outdoors temperatures could explain trends observed though the months, whereas changes on tDFI values derived from sperm incubation at 37°C could explain differences observed between semen samples incubated during 24 and 48 h.

In this work we have observed an increase of sperm DNA fragmentation levels depending on Tave, Tmax and THI mainly in the period between 29 to 35 days bsc, but also for the days 7 to 14. A lower magnitude effect was found for days 37 to 42 and 45 to 47. However, no relationship was found between environmental parameters existing at collection date and DFI parameters. Spermatogenesis in ovine is 49 days long, plus 11 days of sperm maturation at epididymus [53]. Considering day zero that of sperm collection, the period comprised between 7 to 14 days bsc must correspond to the early stages of epididymal maturation; the period comprised between 29 to 35 days bsc coincide with the meiosis I and II processes in which primary spermatocytes (2n) change to secondary spermatocytes (n); the period comprised between 37 to 42 days bsc would correspond to an intermediate period between the mitosis and meiosis phases; and the period comprised between 45 to 47 days bsc would be part of the spermatogenesis process when dormant spermatogonium type A2 evolve to active spermatogonium type A3 and begin the mitosis process (spermatocytogenesis) [54].

An association between the genotype of the SNP located at position −660 in the promoter of the *HSP90AA1* gene, Temperature/THI over the estimated thresholds and sperm DNA fragmentation levels have been observed which was higher for the time periods comprised from 7 to 14 and from 29 to 35 days bsc. Thus, animals carrying the CC_−660_ genotype showed quite similar values of xDFI and tDFI than those with the CG_−660_, and both showed lower levels than males carrying the GG_−660_ genotype (up to 1.7 times) when environmental temperatures exceeds a threshold over 22°C and 29°C for Tave/THI and Tmax, respectively. In a previous work [48], higher expression levels of the *HSP90AA1* gene, in these same rams were observed for animals carrying the CC_−660_ genotype that those with the CG_−660_ (FC (Fold change)  = 1.20) and the GG_−660_ (FC = 1.22) genotypes, when blood samples were collected in August at 34.4°C of maximum environmental temperature. Thus, the lower expression rates found for the GG_−660_ animals under a heat stress environment could be associated to higher values of sperm DNA fragmentation. Also these results pointed out that critical steps of the spermatogenesis process regarding heat-stress susceptibility may be occurring at periods 7 to 14 and 29 to 35 days bsc.

Meiosis is thought to be followed by a brief period of high transcriptional activity, and a large number of mRNAs are stored as messenger ribonucleoprotein particles [55]. Messenger RNA sequestration provides a mechanism by which mRNAs can be synthesized prior to transcriptional arrest and translated when their protein product is required [56]. Most stress-response genes are regulated in a concordant manner with respect to transcript levels and translational efficiency. A strong overall correlation has been observed between transcriptional/translational induction of genes and translation of the corresponding proteins [57]. Therefore, differences in the expression level of the *HSP90AA1* gene will be accompanied by differences in the amount of protein produced. High temperatures (or THI) existing between 29–35 days bsc would activate the heat shock response increasing the *HSP90AA1* gene transcription/translation. Suboptimal expression rates of the *HSP90AA1* gene at this spermatogenesis step would lead to meiosis failures and defects of histone DNA packing. In this sense, in a study to ascertain the program whereby histones are handled before incorporation onto chromatin, a complex containing HSP90, tNASP, histone H4 and histone eH3.1 was found in the cytosolic fraction (S100) [39] Campos et al. [58] suggest that tNASP is a HSP90 co-chaperone for the assembly of the H3.1-H4 units. Therefore the efficiency of the traslocation of histones to the nucleus seems to depend on the available amounts of HSP90 to form the tNASP-HSP90 complex.

The period comprised between days 7 to 14 bsc may coincide with the first steps of the spermiogenesis process in which histones are replaced by protamines. Heat stress at this time point could produce an incomplete DNA protamination if no proper machinery to avoid heat effects is available, making spermatozoa more vulnerable to attack by endogenous and exogenous agents [59], [60], [61]. DNA fragmentation is more frequent in protamine deficient spermatozoa [62]. High number of *HSP90AA1* transcripts present at this step will yield more Hsp90α protein amounts to cope with heat stress effects over protein denaturation and missfolding.

In this breed we have observed a low frequency of the GG_−660_ genotype (<10%) in the milk progeny tested rams (424 rams genotyped; data not shown). Frequencies of CC_−660_ and CG_−660_ genotypes were 43% and 42%, respectively. We have confirmed that there is no association between the estimated breeding values (EBVs) for milk yield and the G/C_−660_ genotype of these rams. Since animals are selected based on their genetic merit for milk production, the low frequency of GG_−660_ males will be due to other causes not directly related to the production level. Thus, we could hypothesize that the GG_−660_ males would have a smaller reproductive capacity, which leave less offspring and this will lead to a lower frequency of GG_−660_ genotype. The presumed lower fertility of the GG_−660_ males could be due to their lesser degree of resistance to thermal stress. Thus, exposure to heat can lead to increased levels of DNA fragmentation, and therefore, a decrease of fertility, as observed in this study. Moreover, results from the present study suggest that highest DNA fragmentation occurs in the late summer. For short-day breeders, with favorable breeding season starting in September, the consequences of heat stress on breeding may be more significant.

Recently, Nordstoga et al. [23] showed an association between sperm DNA integrity and the non returned rate among Norwegian cross-bred rams. In this sense, future works must be directed to assess if changes observed in DFI values of rams with alternative genotypes of the G/C_−660_ mutation at the *HSP90AA1* gene promoter have effect on their fertility. Since rams used in this experiment belongs to the progeny test of the milk breeding program of Manchega sheep breed, many records of insemination results are available. With appropriate statistical models, we would assess if there are differences in the pregnancy rate of rams with alternative genotypes for the G/C_−660_ transversion when sperm samples are used in the AI and they could have been subjected to heat stress in some critical steps of the spermatogenesis process. As Hsp90 genes are highly conserved, in structure and function, in mammals, this study can be extended to other species to test if their reproductive efficiency is compromised by polymorphisms affecting expression rate and/or protein sequence of these genes.

## Materials and Methods

### Ethics Statement

The current study was carried out under a Project License from the INIA Scientific Ethic Committee. Animal manipulations were performed according to the Spanish Policy for Animal Protection RD 53/2013, which meets the European Union Directive 86/609 about the protection of animals used in experimentation. We hereby confirm that the INIA Scientific Ethic Committee, which is the named IACUC for the INIA, specifically approved this study. Animals belong to an artificial insemination centre, were raised in small groups in different barns and fed according to their necessities.

### Animals

A total of 60 adult rams from Manchega dairy sheep breed were used in this study. Males were kept at the Regional Centre of Animal Selection and Reproduction (CERSYRA) in Valdepeñas (Spain) at the same environmental conditions. All males were trained for semen collection by artificial vagina and maintained a regimen of regular collection. All animal handling was done following Spanish Animal Protection Regulation RD 53/2013 which meets the European Union Directive 86/609 about the protection of animals used in experimentation.

### Weather data

Castilla-La Mancha is a region located in the south of Spain which is characterized by an arid environment with a low rainfall and high temperatures. Meteorological data was provided by the Irrigation Advisory Service for Farmers (SIAR) in Castilla-La Mancha. The meteorological data set consisted of hourly measures of temperature (°C) and relative humidity (%) on 245 days from March to October 2010. Daily average temperature (Tave, °C), daily maximum temperature (Tmax, °C) and daily average relative humidity (RH, %) were calculated from these hourly records. A temperature-humidity index (THI) was also calculated as proposed by Marai et al. [49] by combining daily average temperature (Tave) in °C with daily average relative humidity (RH) %·0.01:
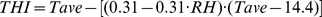



To better understand THI values, Marai et al. [49] proposed a scale of the effect of THI values over heat stress. Thus, values of THI <22.2 assume absence of heat stress; 22.2≥ THI <23.3 means moderate heat stress; 23.3≤ THI <25.6, severe heat stress; and THI ≥25.6 indicates extreme severe heat stress.

### Semen Samples collection

Semen collection was made by artificial vagina. A total of 7 collections per male were carried out, from March to October as a way to ensure that sperm analyses were conducted in different weather conditions of temperature and humidity. After collection, sperm samples were diluted in phosphate-buffered saline (PBS; pH 7.5, 310 mOsm/kg) with 0.5% bovine serum albumin and then incubated in a saline medium at 37°C during 48 hours. The sperm chromatin stability was assessed after collection (0 h) and after 24 and 48 h. Sperm incubation at 37°C has the aim to mimic the environmental circumstances existing at ewe reproductive track.

### DFI assessment

Chromatin stability was assessed by using the Sperm Chromatin Structure Assay (SCSA) technique (SCSA Diagnostics, Inc., Brookings, SD, USA) [63], [64]. This technique is based on the susceptibility of the sperm DNA to acid-induced denaturation *in situ* and in the Acridine Orange (AO) metachromatic acid nucleic staining. This stain fluoresces green when combined with double stranded DNA and red when combined with single stranded DNA (denatured). This technique has been used in rams with good results [52], [65]. García-Álvarez et al. [65] provides a more in depth explanation of the procedure used to assess the sperm chromatin stability in Manchega rams. Briefly, sperm samples were diluted with TNE (0.15 M NaCl, 0.01 M Tris HCl, 1 mM EDTA; pH 7.4) buffer at a final sperm concentration of 2×10^6^ cells/ml and flash frozen in LN2 and stored at −80°C until analysis. For the analysis, the samples were thawed on crushed ice, and 200 µl were put on a cytometry tube. Immediately, 400 µl of an acid-detergent solution (0.08 N HCl, 0.15 M NaCl, 0.1% Triton ×100; pH 1.4) were added to the tube. After exactly 30 s, 1.20 ml of Acridine Orange (AO)-staining solution (0.037 M citric acid, 0.126 M Na_2_HPO_4_, 0.0011 M dissodium EDTA, 0.15 M NaCl; pH 6.0, 4°C) containing 6 mg/ml electrophoretically purified AO was added. Stained samples were analyzed just after 3 minutes by flow cytometry, being the excited AO used as the fluorophore. AO was excited by using an argon laser providing 488 µm light. A total of 5000 events were accumulated for each sample. We have expressed the extent of DNA denaturation in terms of DNA Fragmentation Index (DFI), which is the ratio of red to total (red plus green) fluorescence intensity, i.e. the level of denatured DNA over the total DNA [64]. The DFI value was calculated for each sperm cell in a sample, and the resulting DFI frequency profile was obtained. Each sperm sample was characterized by a mean (xDFI) and a standard deviation (sdDFI). Total DNA fragmentation index (tDFI) was defined as the percentage of spermatozoa with a DFI value over 25.

### HSP90AA1 genotypes

The 60 adult rams of Manchega breed used in this study were selected on the basis of its genotype for the transversion G/C located at position -660 in the promoter region of the *HSP90AA1* gene (SNPs have been recently submitted to the NCBI dbSNP. Actual information GeneBank acc. number DQ983231.1). These animals proceed from a previous work [48] focused on the study of expression differences of alternative genotypes of seven SNPs located in the *HSP90AA1* promoter. Twenty males of each genotype CC_−660_, CG_−660_ and GG_−660_ were selected, and used in this work to assess sperm DNA fragmentation.

### Statistical analysis

The present study consisted of two steps: in a first step, we studied the effect of temperature prior to semen collection on the degree of sperm DNA fragmentation. For that, measures of Tave, Tmax and THI from the day 60 prior to semen collection to date of collection were used. The goal was to identify which days had a significant effect on DFI values. In a second step, we studied whether there is an association between DFI values recorded under differential climatic conditions and the genotype of the SNP located in the *HSP90AA1* gene promoter.

To examine the effect of weather conditions on the degree of DFI of spermatozoa in rams, a Ridge Regression analysis including the Tave, Tmax and THI measures of all days from the day of semen collection to 60 days previous to semen collection (period in which spermatogenesis must be developed) were performed. The high correlation among temperature measures of consecutive days leads to problems of multicollinearity. Ridge Regression analysis [66] is a kind of penalized least squares procedure which is recommended when predictor variables in a multiple regression model are highly correlated. Applying the Ridge Regression penalty in the analysis, it has the effect of shrinking the estimated regression coefficients toward zero, reducing the variance of the estimate. The model used for the Ridge Regression was the following:
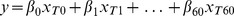



The ridge regression estimator of β is:
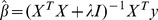
where, *y* is the vector of DFI measures (xDFI, sdDFI and tDFI) corrected by some environmental effects, *X* is a matrix containing the Tave, Tmax or THI measures from the day 60 previous to semen collection to date of the collection, *β* the vector of regression coefficients, and λ the shrinkage parameter. Given that sperm DFI values could be affected by various environmental effects, DFI values were corrected by the time of incubation (0, 24 and 48 h) and the month of semen collection prior to Ridge Regression analysis. Collection month was considered as a way of handling management differences observed throughout the year. No other effects such us housing or age of males were considered as were the same for all males. The value of λ may range from 0 to +∞. If λ = 0, ridge regression estimates are equal to ordinary least squares (OLS) estimates; if λ = +∞, ridge regression estimates are equal to 0. Cross-validation was used to find the best value of λ. Ridge Regression analysis was carried out using the functions available in the MASS package [67] and the glmnet package [68] of the R statistical software [69].

Once the days having significant effect on the degree of sperm DNA fragmentation were identified, we examined whether males with different genotype for the SNP located in the *HSP90AA1* gene promoter may have a differential response in the associated variation of DFI to heat stress. A linear mixed model including genotype, sperm incubation time (0 h, 24 h and 48 h), a summary measure for the Tave, Tmax or THI and its interactions as fixed effects, and male as random effect were fitted. As summary weather measures we considered the average and maximum for Tave, Tmax and THI for the days prior to semen collection that showed a significant effect on the ridge regression analysis. Since it was expected that the effect of temperature on DFI values was revealed from a threshold, a piecewise linear function was used to model the temperature effect:
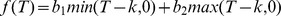
where, *T* is the Tave, Tmax or THI measure and *k* is the selected threshold. As threshold we used values ranging from 15 to 35, a range in which we expected to find the threshold above which DFI values increased, and retained the value that best fit the data. Heterogeneous residual variance for the effect of sperm incubation time was considered. Several models consisting of combinations of effects were compared by maximum likelihood (MLE). The model that showed better fit (data not shown) was the following:
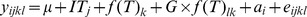
where:*y_ijkl_*: DFI measure (xDFI and tDFI)

µ: global mean

*IT_j_*: sperm incubation time (3 levels: 0, 24 and 48 h)

f(T)_k_: effect of temperature measure (Tave, Tmax and THI) for the days prior sperm collection showing a significant effect.

*G*×f*(T)_lk_*: *HSP90AA1* genotype (3 levels: CC, CG and GG) – temperature effect interaction

*a_i_*: male (60 levels)

*e_ijkl_*: heterogeneous random residual error ∼ N(0, σ_e*i*_^2^)

Statistical analysis was performed using the libraries *nlme* and *lme4* from the R-statistical analysis package [70]. Differences between *HSP90AA1* genotypes on the effect of the T^a^/THI on the sperm DFI values were tested using the *multcomp* package [71].

## Supporting Information

Figure S1 Regression coefficients from the mixed-effects model relating DFI values with summary measure of Tave, Tmax and THI for the days 37 to 42 and 45 to 47 before semen collection For each coefficient in the model, estimates (points) plus and minus 1 (bold line) and 2 (thin line) standard deviations are represented. ^*^
(DOC)

Table S1 Summary of mixed model effects relating DFI values with Tave, Tmax and THI for the days 7 to 14 prior to semen collection.^*^
(DOC)

Table S2 Summary of mixed model effects relating DFI values with Tave, Tmax and THI for the days 29 to 35 prior to semen collection.^*^
(DOC)

Table S3 Summary of mixed model effects relating DFI values with Tave, Tmax and THI for the days 37 to 42 prior to semen collection.^*^
(DOC)

Table S4 Summary of mixed model effects relating DFI values with Tave, Tmax and THI for the days 45 to 47 prior to semen collection.^*^
(DOC)

## References

[1] Araújo MB, Rahbek C (2006) How does climate change affect biodiversity? Science 8: 1396–1397. 10.1126/science.1131758 16959994

[2] Araújo MB, Alagador D, Cabeza M, Nogues-Bravo D, Thuiller W (2011) Climate changes threatens European conservation areas. Ecology letters 14: 484–492. 21447141 10.1111/j.1461-0248.2011.01610.x PMC3116148

[3] Thomas CD, Cameron A, Green RE, Bakkenes M, Beaumont LJ (2004) Extinction risk from climate change. Nature 8: 14–148. 10.1038/nature02121 14712274

[4] Grazer VM, Martin OY (2012) Investigating Climate Change and Reproduction: Experimental Tools from Evolutionary Biology. Biology 1: 411–438. 24832232 10.3390/biology1020411 PMC4009780

[5] Groen AF, Steine T, Colleau JJ, Pedersen J, Pribyl J, et al (1997) Economic values in dairy cattle breeding, with special reference to functional traits. Repost of an EAAP-working group. Livest Prod Sci 49: 1–21.

[6] Legarra A, Ramon M, Ugarte E, Pérez-Guzmán MD (2007) Economic weights of fertility, prolificacy, milk yield and longevity in dairy sheep. Animal 1: 193–203. 22444284 10.1017/S1751731107657814

[7] Jannes P, Spiessens C, Van der Auwera I, D'Hooghe T, Verhoeven G, et al (1998) Male subfertility induced by acute scrotal heating affects embryo quality in normal female mice. Hum Reprod 13: 372–375. 9557841 10.1093/humrep/13.2.372

[8] Pérez-Crespo M, Pintado B, Gutierrez-Adán A (2008) Scrotal heat stress effects on sperm viability, sperm DNA integrity and the Offspring sex ratio in mice. Molecular Reproduction and Development 75: 40–47. 17474098 10.1002/mrd.20759

[9] Yaeram J, Setchell BP, Maddocks S (2006) Effect of heat stress on the fertility of male mice in vivo and in vitro. Reprod Fertil Dev 18: 647–653. 16930511 10.1071/rd05022

[10] Fleming JS, Yu F, McDonald RM, Meyers SA, Montgomery GW, et al (2004) Effects of scrotal heating on sperm surface protein PH-20 expression in sheep. Mol Reprod Dev 68: 103–114. 15039954 10.1002/mrd.20049

[11] Sailer BL, Sarkar LJ, Jost LK, Bjordahl J, Evenson DP (1997) Effects of heat stress on mouse testicular cells and sperm chromatin structure as measured by flow cytometry. J Androl 18: 294–301. 9203058

[12] Banks S, King SA, Irvine DS, Saunders PTK (2005) Impact of a mild scrotal heat stress on DNA integrity in murine spermatozoa. Reproduction 129: 505–514. 15798026 10.1530/rep.1.00531

[13] Conwell CC, Vilfan ID, Hud NV (2003) Controlling the size of nanoscale toroidal DNA condensates with static curvature and ionic strength. Proc Natl Acad Sci USA 100: 9296–9301. 12871999 10.1073/pnas.1533135100 PMC170912

[14] Barratt CL, Aitken RJ, Björndahl L, Carrell DT, de Boer P, et al (2010) Sperm DNA: Organization, protection and vulnerability: From basic science to clinical applications-A position report. Hum Reprod 25: 824–838. 20139429 10.1093/humrep/dep465

[15] González-Marín C, Gosálvez J, Roy R (2012) Types, causes, detection and repair of DNA fragmentation in Animal and Human sperm cells. Int J Mol Sci 13: 14026–14052. 23203048 10.3390/ijms131114026 PMC3509564

[16] Evenson DP, Wixon R (2006) Clinical aspects of sperm DNA fragmentation detection and male fertility. Theriogenology 65: 979–991. 16242181 10.1016/j.theriogenology.2005.09.011

[17] Amann RP, DeJarnette JM (2012) Impact of genomic selection on AI dairy sires on their likely utilization and methods to estimate fertility: A paradigm shift. Theriogenology 77: 795–817. 22153268 10.1016/j.theriogenology.2011.09.002

[18] Evenson DP, Jost LK, Zinaman MJ, Clegg E, Purvis K, et al (1999) Utility of the sperm chromatin structure assay (SCSA) as a diagnostic and prognostic tool in the human fertility clinic. Hum Reprod 14: 1039–1049. 10221239 10.1093/humrep/14.4.1039

[19] Spanò M, Bonde JP, Hjollund HI, Kolstad HA, Cordelli E, et al (2000) Sperm chromatin damage impairs human fertility. The Danish First Pregnancy Planner Study Team. Fertil Steril 73: 43–50. 10632410 10.1016/s0015-0282(99)00462-8

[20] Bungum M, Humaidan P, Spanò M, Jepson K, Bungum L, et al (2004) The predictive value of sperm chromatin structure assay (SCSA) parameters for the outcome of intrauterine insemination, IVF and ICSI. Hum Reprod 19: 1401–1408. 15117894 10.1093/humrep/deh280

[21] Ballachey BE, Hohenboken WD, Evenson DP (1987) Heterogeneity of sperm nuclear chromatin structure and its relationship to fertility of bulls. Biology of Reproduction 36: 915–925. 3593856 10.1095/biolreprod36.4.915

[22] Bradley AD, Kasperson KM, Wixon RL, Evenson DP (2009) Boar Fertility and Sperm Chromatin Structure Status: A Retrospective Report. Journal of Andrology 30: 1–6. 19478334 10.2164/jandrol.108.006254

[23] Nordstoga A, Krogenæs A, Nødtvedt A, Farstad W, Waterhouse K (2013) The Relationship Between Post-Thaw Sperm DNA Integrity and Non-Return Rate Among Norwegian Cross-Bred Rams. Reprod Dom Anim 48: 207–212. 10.1111/j.1439-0531.2012.02132.x 22882422

[24] Ulberg LC (1958) The influence of high temperature on reproduction. J Hered 49: 62–64.

[25] Hales BF, Aguilar-Mahecha A, Robaire B (2005) The stress response in gametes and embryos after paternal chemical exposures. Toxicol Appl Pharmacol 207: 514–520. 15982682 10.1016/j.taap.2004.12.021

[26] De Vita R, Calugi A, Chiarantano C, Forte D, Mauro F, et al (1990) Effects of heat on mouse spermatogenesis monitored by flow cytometry. Int J Hypertherm 6: 543–551. 10.3109/02656739009140950 2376667

[27] Paul C, Murray AA, Spears N, Saunders PTK (2008) A single, mild, transient scrotal heat stress causes DNA damage, subfertility and impairs formation of blastocysts in mice. Reproduction 136: 73–84. 18390691 10.1530/REP-08-0036

[28] Setchell BP (2006) The effects of heat on the testes of mammals. Anim Reprod 3: 81–91.

[29] Biggiogera M, Tanguay RM, Marin R, Wu Y, Martin TE, et al (1996) Localization of heat shock proteins in mouse male germ cells: an immunoelectron microscopical study. Exp Cell Res 229: 77–85. 8940251 10.1006/excr.1996.0345

[30] Yin Y, DeWolf WC, Morgentaler A (1998) Experimental cryptorchidism induces testicular germ cell apoptosis by p53-dependent and -independent pathways in mice. Biol Reprod 58: 492–496. 9475406 10.1095/biolreprod58.2.492

[31] Itoh H, Tashima Y (1990) A novel testis-specific 105-kDa protein related to the 90-kDa heat-shock protein. Eur J Biochem 193: 429–435. 2226463 10.1111/j.1432-1033.1990.tb19356.x

[32] Itoh H, Tashima Y (1991) Different expression time of the 105-kDa protein and 90-kDa heat-shock protein in rat testis. FEBS Lett 289: 110–112. 1893998 10.1016/0014-5793(91)80920-x

[33] Zhang XS, Lue YH, Guo SH, Yuan JX, Hu ZY, et al (2005) Expression of HSP105 and HSP60 during germ cell apoptosis in the heat-treated testes of adult cynomolgus monkeys (*Macaca fascicularis*). Front Biosci 10: 3110–3121. 15970565 10.2741/1767

[34] Sørensen JG, Kristensen TN, Loeschcke V (2003) The evolutionary and ecological role of heat shock proteins. Ecol Lett 6 6: 1025–1037.

[35] Sreedhar AS, Kalmár E, Csermely P, Shen YF (2004) Hsp90 isoforms: functions, expression and clinical importance. FEBS Lett 562: 11–15. 15069952 10.1016/s0014-5793(04)00229-7

[36] Vamvakopoulos NC (1993) Tissue-specific expression of heat shock protein 70 and 90. Potential implication for differential sensitivity of tissues to glucocorticoids. Mol Cell Endocrinol 98: 49–54. 8143913 10.1016/0303-7207(93)90235-c

[37] Yue L, Karr TL, Nathan DF, Swift H, Srinivasan S, et al (1999) Genetic analysis of viable Hsp90 alleles reveals a critical role in Drosophila spermatogenesis. Genetics 151: 1065–1079. 10049923 10.1093/genetics/151.3.1065 PMC1460532

[38] Grad I, Cederroth CR, Walicki J, Grey C, Barluenga S, et al.. (2010) The Molecular Chaperone Hsp90α Is Required for Meiotic Progression of Spermatocytes beyond Pachytene in the Mouse. Plos One 12. 10.1371/journal.pone.0015770 PMC3013136 21209834

[39] Alekseev OM, Widgren EE, Richardson RT, O'Rand MG (2005) Association of NASP with HSP90 in mouse spermatogenic cells. Stimulation of ATPase activity and transport of linker histones into nuclei. J Biol Chem 280: 2904–2911. 15533935 10.1074/jbc.M410397200

[40] Meistrich ML, Bucci LR, Trostle-Weige PK, Brock WA (1985) Histone variants in rat spermatogonia and primary spermatocytes. Developmental Biology 112: 230–340. 3932111 10.1016/0012-1606(85)90137-x

[41] Drabent B, Bode C, Miosge N, Herken R, Doenecke D (1998) Expression of the mouse histone gene H1t begins at premeiotic stages of spermatogenesis. Cell Tissue Res 291: 127–132. 9394050 10.1007/s004410050986

[42] Grimes SR, Wilkerson DC, Noss KR, Wolfe SA (2003) Transcriptional control of the testis-specific histone H1t gene. Gene 304: 13–21. 12568711 10.1016/s0378-1119(02)01201-5

[43] Alekseev OM, Bencic DC, Richardson RT, Widgren EE, O'Rand MG (2003) Overexpression of the Linker histone-binding protein tNASP affects progression through the cell cycle. J Biol Chem 278: 8846–8852. 12509435 10.1074/jbc.M210352200

[44] Marcos-Carcavilla A, Calvo JH, González C, Moazami-Goudarzi K, Laurent P, et al (2008) Structural and functional analysis of the *HSP90AA1* gene: distribution of polymorphisms among sheep with different responses to scrapie. Cell Stress and Chaperones 13: 19–29. 18347938 10.1007/s12192-007-0004-2 PMC2666211

[45] Öner Y, Calvo JH, Serrano M, Elmaci C (2012) Polymorphisms at the 5′ flanking region of the *HSP90AA1* gene in native Turkish sheep breeds. Livest Sci 50: 381–385.

[46] Marcos-Carcavilla A, Moreno C, Serrano M, Laurent P, Cribiu EP, et al (2010) Polymorphisms in the *HSP90AA1* 5′ flanking region are associated with scrapie incubation period in sheep. Cell Stress and Chaperones 15: 343–349. 19838832 10.1007/s12192-009-0149-2 PMC3082647

[47] Marcos-Carcavilla A, Mutikainen M, González C, Calvo JH, Kantanen J, et al (2010) A SNP in the *HSP90AA1* gene 5′ flanking region is associated with the adaptation to differential thermal conditions in the ovine species. Cell Stress and Chaperones 15: 67–81. 19496025 10.1007/s12192-009-0123-z PMC2866970

[48] Salces-Ortiz J, González C, Moreno-Sánchez N, Calvo JH, Pérez-Guzmán MD, et al. (2013) Ovine *HSP90AA1* Expression Rate Is Affected by Several SNPs at the Promoter under Both Basal and Heat Stress Conditions. Plos One 8.. 10.1371/journal.pone.0066641 PMC3691178 23826107

[49] Marai IFM, El-Darawany AA, Fadiel A, Abdel-Hafez MAM (2007) Physiological traits as affected by heat stress in sheep - a review. Small Rum Res 71: 1–12.

[50] Fernandez-Gonzalez R, Moreira PN, Perez-Crespo M, Sanchez-Martin M, Ramirez MA, et al (2008) Long-term effects of mouse intracytoplasmic sperm injection with DNA-fragmented sperm on health and behavior of adult offspring. Biol Reprod 78: 761–772. 18199884 10.1095/biolreprod.107.065623

[51] Delbès G, Hales BF, Robaire B (2010) Toxicants and human sperm chromatin integrity. Mol Hum Reprod 16: 14–22. 19812089 10.1093/molehr/gap087

[52] Garcia-Macias V, Martinez-Pastor F, Alvarez M, Borragan S, Chamorro CA, et al (2006) Seasonal changes in sperm chromatin condensation in ram (*Ovis aries*), red deer (*Cervus elaphus*) and brown bear (*Ursus arctos*). J Androl 27: 837–846. 16837731 10.2164/jandrol.106.000315

[53] Amir D, Ortavant R, Bure A (1968) Influence de la fréquence des collectes sur la durée du transit des spermatozoïdes dans le canal épididymaire du bélier. Ann Biol Anim Biochim Biophys 8: 195–207.

[54] Cardoso FM, Queiroz GF (1988) Duration of the cycle of the seminiferous epithelium and daily sperm production of Brazilian hairy rams. Anim Reprod Sci 17: 77–84.

[55] Hecht N (1998) Molecular mechanisms of male germ cell differentiation. Bioessays 20: 555–561. 9723004 10.1002/(SICI)1521-1878(199807)20:7<555::AID-BIES6>3.0.CO;2-J

[56] Hecht N (2000) Intracellular and intercellular transport of many germ cell mRNAs is mediated by the DNA- and RNA-binding protein, testis-brain-RNA-binding protein (TB-RBP). Mol Reprod Dev 56: 252–253. 10824978 10.1002/(SICI)1098-2795(200006)56:2+<252::AID-MRD8>3.0.CO;2-8

[57] Lackner DH, Schmidt MW, Wu SD, Wolf DA, Bahler J (2012) Regulation of transcriptome, translation, and proteome in response to environmental stress in fission yeast. Genome Biology 13. 10.1186/gb-2012-13-4-r25 PMC3446299 22512868

[58] Campos EI, Fillingham J, Li G, Zheng H, Voigt P, et al (2010) The program for processing newly-synthesized histones H3.1 and H4. Nat Struct Mol Biol 17: 1343–1351. 20953179 10.1038/nsmb.1911 PMC2988979

[59] Szczygiel MA, Ward WS (2002) Combination of dithiothreitol and detergent treatment of spermatozoa causes paternal chromosomal damage. Biol Reprod 67: 1532–1537. 12390885 10.1095/biolreprod.101.002667

[60] Sotolongo B, Lino E, Ward WS (2003) Ability of hamster spermatozoa to digest their own DNA. Biol Reprod 69: 2029–2035. 12930713 10.1095/biolreprod.103.020594

[61] Oliva R (2006) Protamines and male infertility. Hum Reprod Update 12: 417–435. 16581810 10.1093/humupd/dml009

[62] Nasr-Esfahani MH, Salehi M RS, Anjomshoa M, Rozbahani S, et al (2005) Effect of sperm DNA damage and sperm protamine deficiency on fertilizacion and embryo development post-ICSL. Reprod Biomed Online 9: 652–658. 10.1016/s1472-6483(10)60959-5 16168218

[63] Evenson DP, Jost L (2000) Sperm chromatin structure assay is useful for fertility assessment. Methods Cell Sci 22: 169–189. 11264952 10.1023/a:1009844109023

[64] Evenson DP, Larson KL, Jost LK (2002) Sperm chromatin structure assay: its clinical use for detecting sperm DNA fragmentation in male infertility and comparisons with other techniques. J Androl 23: 25–43. 11780920 10.1002/j.1939-4640.2002.tb02599.x

[65] Garcia-Alvarez O, Maroto-Morales A, Ramon M, del Olmo E, Montoro V, et al (2010) Analysis of selected sperm by density gradient centrifugation might aid in the estimation of in vivo fertility of thawed ram spermatozoa. Theriogenology 74: 979–988. 20580077 10.1016/j.theriogenology.2010.04.027

[66] Hoerl AE, Kennard RW (1970) Ridge Regression. Applications to Non-orthogonal Problems. Technometrics 12: 69–82.

[67] Venables WN, Ripley BD (2002) Modern Applied Statistics with S. Fourth Edition. Springer, New York.

[68] Friedman J, Hastie T, Tibshirani R (2010) Regularization Paths for Generalized Linear Models via Coordinate Descent. Journal of Statistical Software 33: 1–22. 20808728 PMC2929880

[69] R Core Team (2013) A language and environmental for statistical computing. R Foundation for Statistical Computing, Vienna, Austria.

[70] Pinheiro JC, Bates DM (2000) Mixed-Effects Models in S and S-PLUS. Statistics and Computer Series, Springer-Verlag, New York, NY.

[71] Hothorn T, Bretz F, Westfall P (2008) Simultaneous Inference in General Parametric Models. Biometrical Journal 50: 346–363. 18481363 10.1002/bimj.200810425

