# Correction: Influence of the Temperature and the Genotype of the *HSP90AA1* Gene over Sperm Chromatin Stability in Manchega Rams

**DOI:** 10.1371/journal.pone.0095407

**Published:** 2014-04-16

**Authors:** The PLOS ONE Staff

The figures have been corrected for improved readability.

Please see the corrected Figure 1 here.

**Figure 1:**
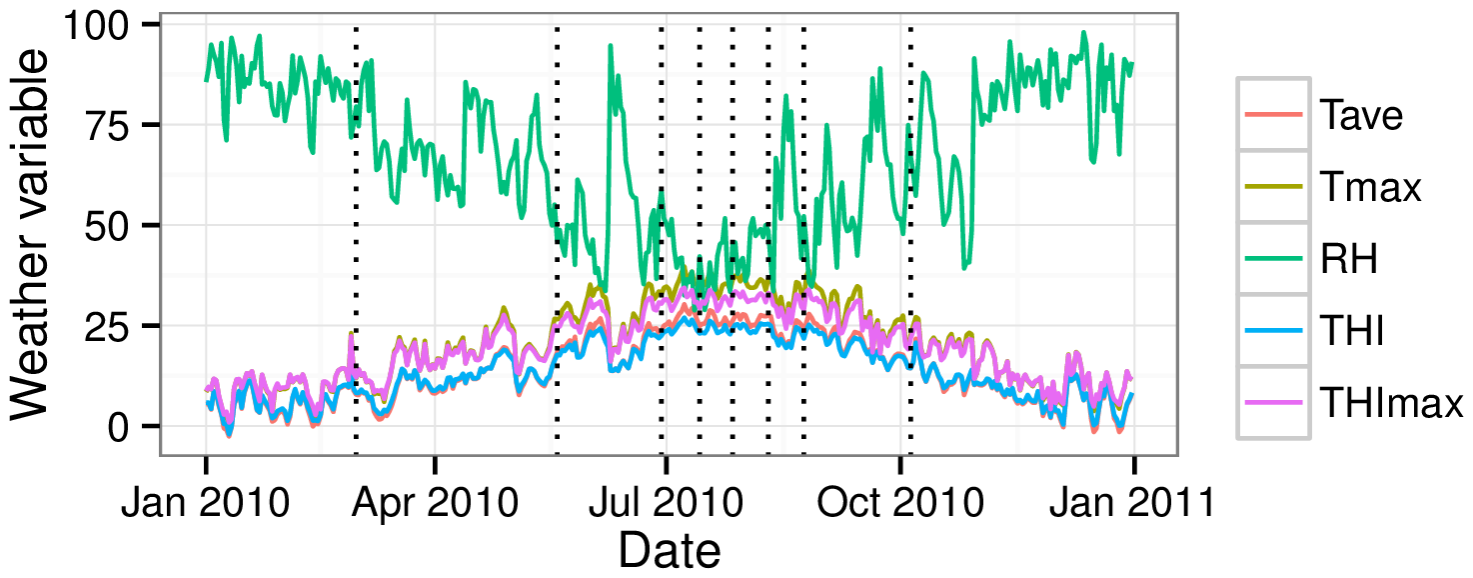
Trends of daily average (Tave, °C) and maximun (Tmax, °C) temperatures, relative humidity (RH, %) and average (THI) and maximum (THImax) temperature humidity index along the year 2010 (Data from SIAR http://crea.uclm.es/siar/datmeteo/). Dotted lines are days of semen collection.

Please see the corrected Figure 2 here.

**Figure 2:**
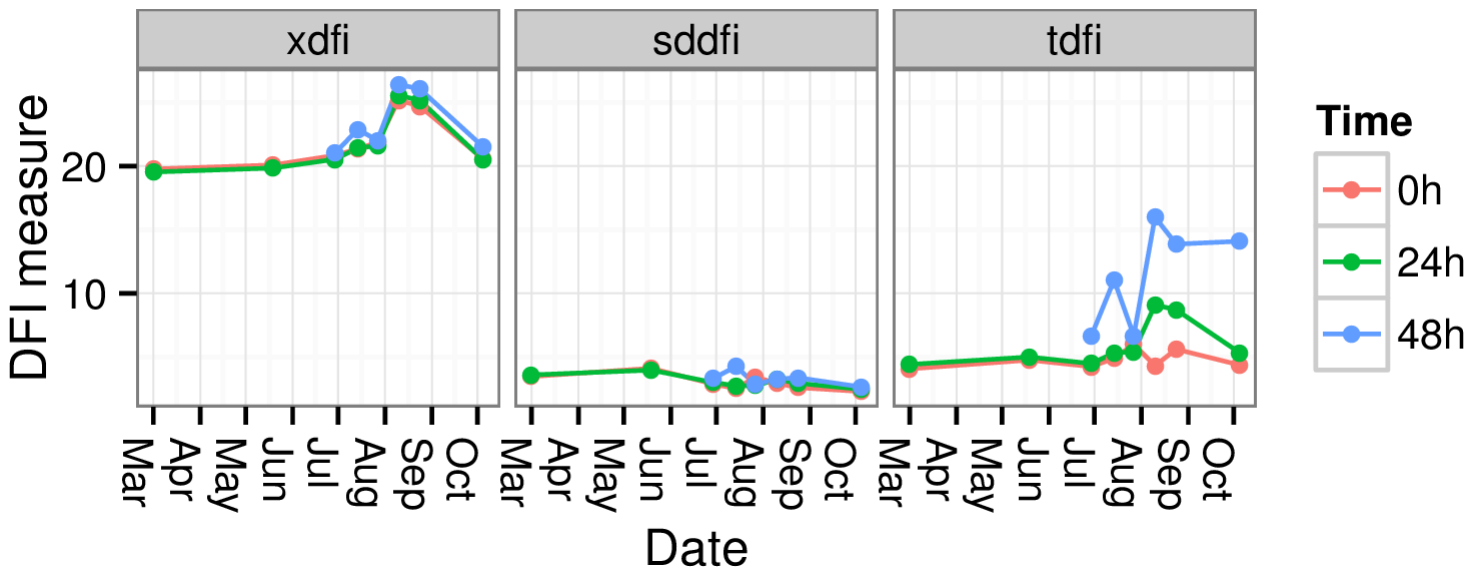
Changes in xDFI, sdDFI and tDFI values with the incubation time (0 h, 24 h and 48 h) along the period of the year from which sperm samples were collected.

Please see the corrected Figure 3 here.

**Figure 3:**
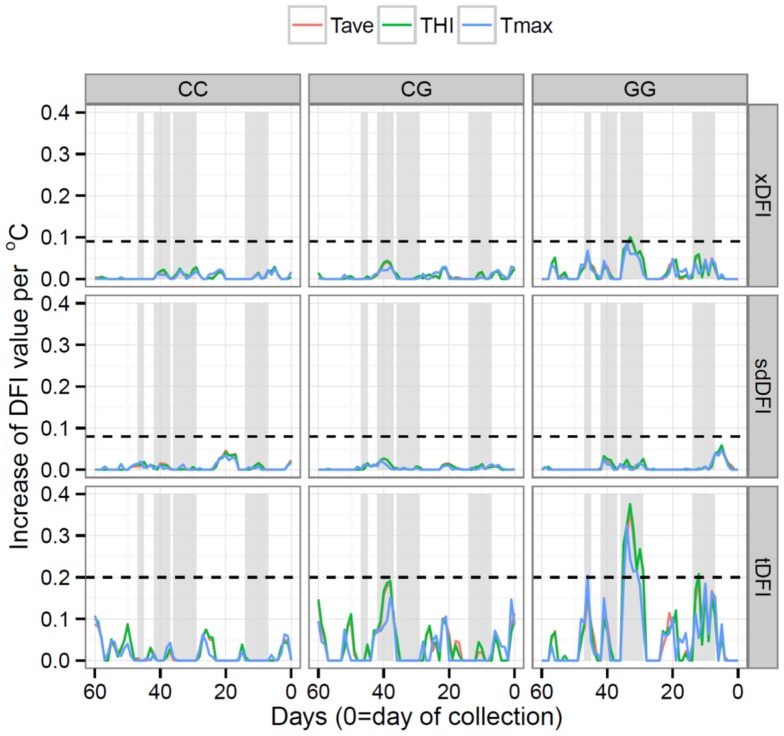
Ridge regression analyses relating DFI measures (xDFI, sdDFI and tDFI) from day 60 prior to semen collection to date of collection with weather measures (Tave  =  average daily temperature, Tmax  =  maximum daily temperature, and THI  =  temperature humidity index), for each HSP90AA1 genotype. Fitted effects extending beyond dotted-lines (---) differ significantly (P<0.05) from zero. Four regions (gray regions) with a significant possible effect on sperm DFI levels were identified.

Please see the corrected Figure 4 here.

**Figure 4:**
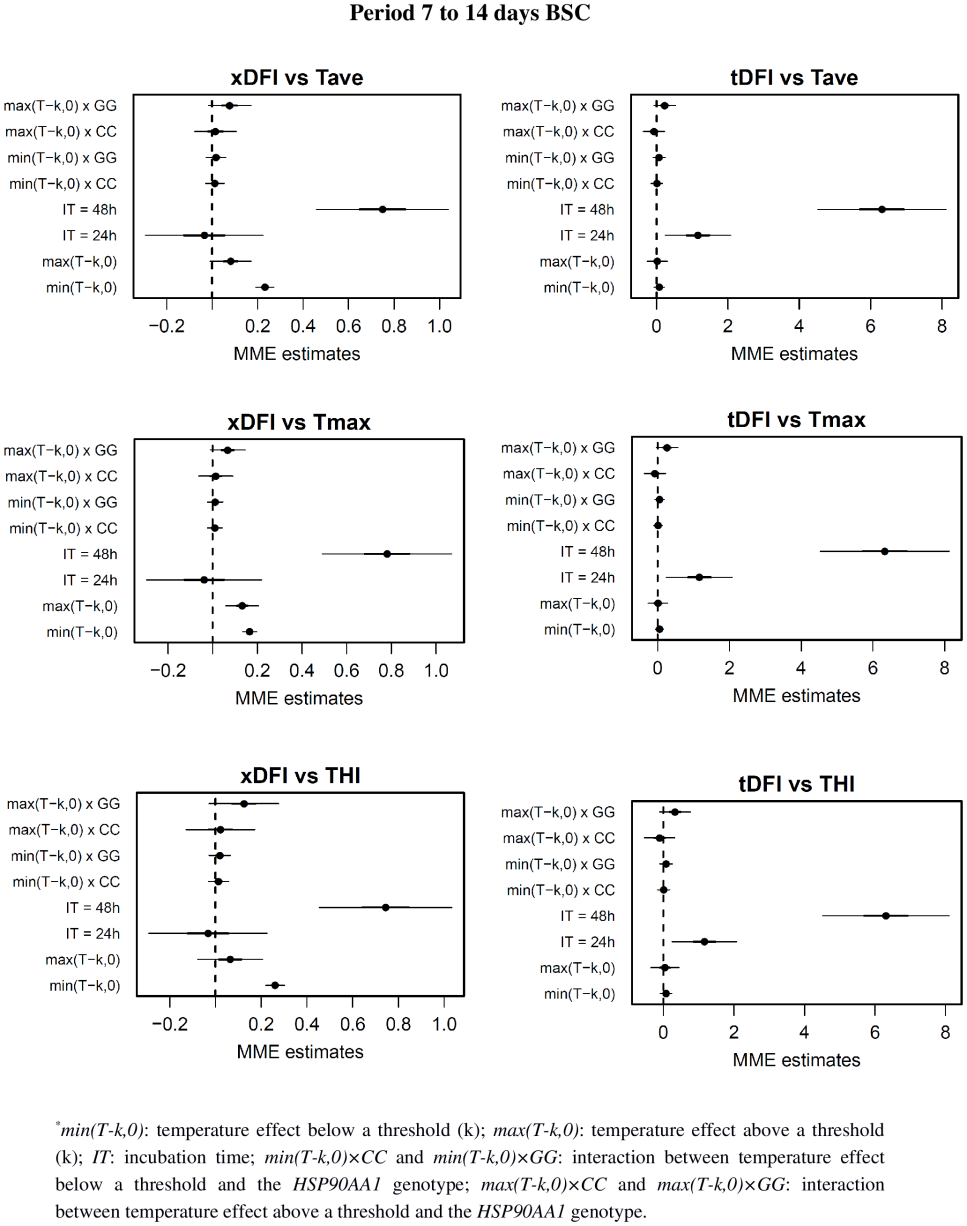
Regression coefficients from the mixed-effects model relating DFI values with summary measure of Tave, Tmax and THI for the days 7 to 14 before semen collection. For each coefficient in the model, estimates (points) plus and minus 1 (bold line) and 2 (thin line) standard deviations are represented. *

Please see the corrected Figure 5 here.

**Figure 5:**
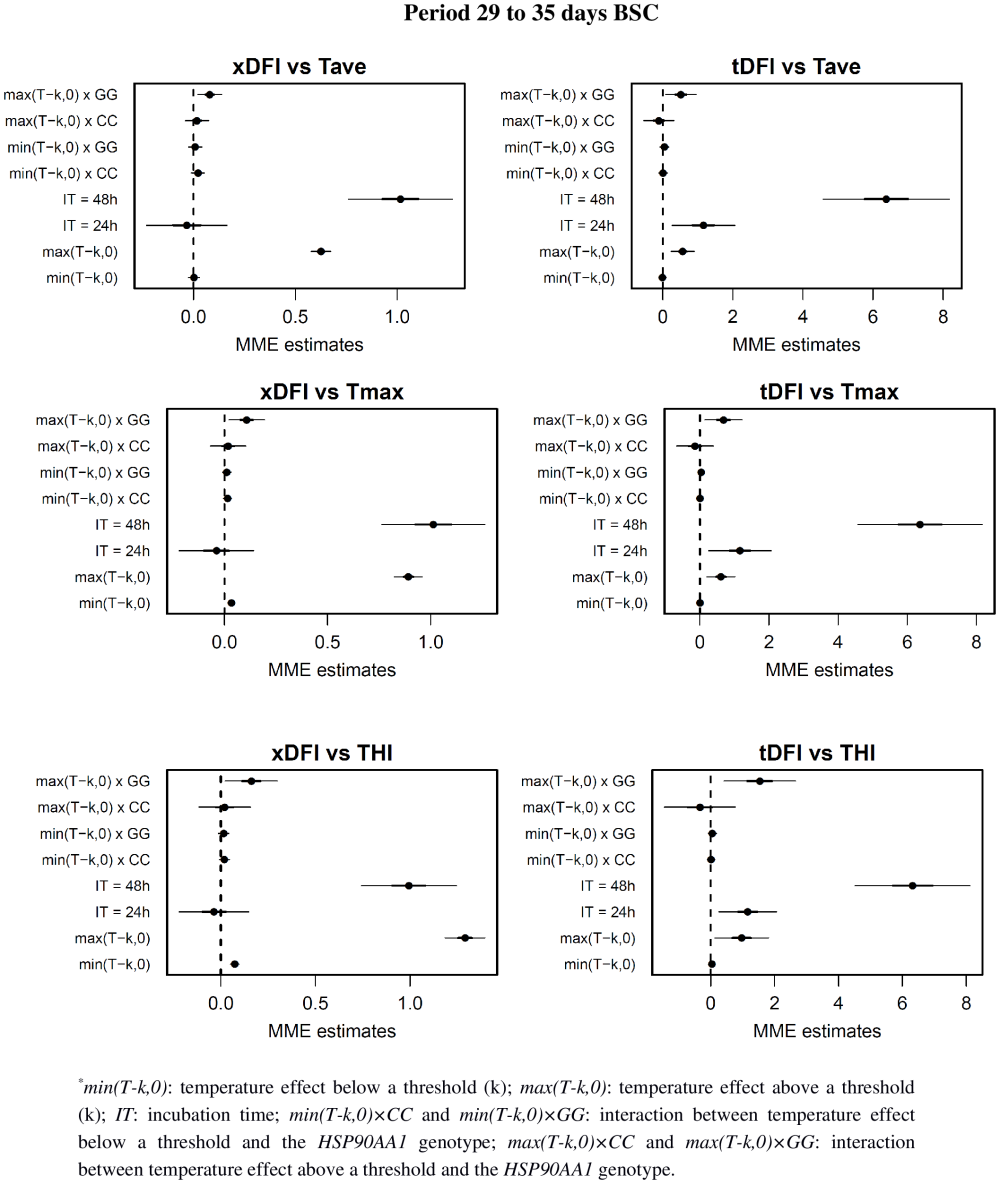
Regression coefficients from the mixed-effects model relating DFI values with summary measure of Tave, Tmax and THI for the days 29 to 35 before semen collection. For each coefficient in the model, estimates (points) plus and minus 1 (bold line) and 2 (thin line) standard deviations are represented. *
