# Supplementary material for: Influence of the Temperature and the Genotype of the HSP90AA1 Gene over Sperm Chromatin Stability in Manchega Rams
Source: PLoS One. 2014 Jan 21;9(1):e86107. doi: 10.1371/journal.pone.0086107 (PMC3897619; doi:10.1371/journal.pone.0086107)
Supplement: Figure S1 — Regression coefficients from the mixed-effects model relating DFI values with summary measure of Tave, Tmax and THI for the days 37 to 42 and 45 to 47 before semen collection For each coefficient in the model, estimates (points) plus and minus 1 (bold line) and 2 (thin line) standard deviations are represented. * (DOC) [file pone.0086107.s001.doc]

**Supplementary Figure S1**. Ramon et al. 2013

| **Period 37 to 42 days BSC** | |  |  | **Period 45 to 47 days BSC** | |
| --- | --- | --- | --- | --- | --- |
|  |  |  |  |  |  |
|  |  |  |  |  |  |
|  |  |  |  |  |  |
|  |  |  |  |  |  |
|  |  |  |  |  |  |
|  |  |  |  |  |  |

^*^*min(T-k,0)*: temperature effect below a threshold (k); *max(T-k,0)*: temperature effect above a threshold (k); *IT*: incubation time; *min(T-k,0)×CC* and *min(T-k,0)×GG*: interaction between temperature effect below a threshold and the *HSP90AA1* genotype; *max(T-k,0)×CC* and *max(T-k,0)×GG*: interaction between temperature effect above a threshold and the *HSP90AA1* genotype.
