# Supplementary material for: Influence of the Temperature and the Genotype of the HSP90AA1 Gene over Sperm Chromatin Stability in Manchega Rams
Source: PLoS One. 2014 Jan 21;9(1):e86107. doi: 10.1371/journal.pone.0086107 (PMC3897619; doi:10.1371/journal.pone.0086107)
Supplement: Table S1 — Summary of mixed model effects relating DFI values with Tave, Tmax and THI for the days 7 to 14 prior to semen collection.* (DOC) [file pone.0086107.s002.doc]

**Table S1.** Summary of mixed model effects relating DFI values with Tave, Tmax and THI for the days 7 to 14 prior to semen collection.^*^

| Parameter | xDFI |  |  |  |  |  |  |  |  |  |  |
| --- | --- | --- | --- | --- | --- | --- | --- | --- | --- | --- | --- |
| Variable | Tave |  |  |  | Tmax |  |  |  | THI |  |  |
|  | Threshold | 22.0˚C | DIC = 5075 |  | Threshold | 28.03˚C | DIC = 5057 |  | Threshold | 21.5 | DIC = 5077 |
|  | Estimate | se | CI95 |  | Estimate | se | CI95 |  | Estimate | se | CI95 |
| Intercept | 22.64 | 0.16 | 22.33 ; 22.95 |  | 21.65 | 0.15 | 21.36 ; 21.95 |  | 22.79 | 0.15 | 22.49 ; 23.06 |
| *min*(T-k,0) | 0.23 | 0.02 | 0.19 ; 0.27 |  | 0.13 | 0.02 | 0.09 ; 0.16 |  | 0.26 | 0.02 | 0.22 ; 0.30 |
| *max*(T-k,0) | 0.08 | 0.05 | -0.00 ; 0.17 |  | 0.21 | 0.03 | 0.16 ; 0.27 |  | 0.06 | 0.07 | -0.08 ; 0.20 |
| IT:24h | -0.03 | 0.13 | -0.29 ; 0.22 |  | -0.04 | 0.13 | -0.30 ; 0.21 |  | -0.03 | 0.13 | -0.27 ; 0.24 |
| IT:48h | 0.75 | 0.15 | 0.46 ; 1.04 |  | 0.83 | 0.15 | 0.54 ; 1.12 |  | 0.74 | 0.15 | 0.47 ; 1.03 |
| *min*(T-k,0) × CC | 0.01 | 0.02 | -0.03 ; 0.05 |  | 0.01 | 0.02 | -0.02 ; 0.05 |  | 0.01 | 0.02 | -0.04 ; 0.05 |
| *min*(T-k,0) × GG | 0.02 | 0.02 | -0.03 ; 0.06 |  | 0.01 | 0.02 | -0.03 ; 0.05 |  | 0.02 | 0.02 | -0.03 ; 0.06 |
| *max*(T-k,0) × CC | 0.01 | 0.05 | -0.08 ; 0.11 |  | 0.01 | 0.03 | -0.05 ; 0.07 |  | 0.02 | 0.08 | -0.13 ; 0.16 |
| *max*(T-k,0) × GG | 0.08 | 0.05 | -0.02 ; 0.17 |  | 0.05 | 0.03 | -0.01 ; 0.10 |  | 0.12 | 0.08 | -0.01 ; 0.29 |
|  |  |  |  |  |  |  |  |  |  |  |  |
| Parameter | tDFI |  |  |  |  |  |  |  |  |  |  |
| Variable | Tave |  |  |  | Tmax |  |  |  | THI |  |  |
|  | Threshold | 21.2˚C | DIC = 8280 |  | Threshold | 30.21 | DIC = 8277 |  | Threshold | 20.6 | DIC = 8282 |
|  | Estimate | se | CI95 |  | Estimate | se | CI95 |  | Estimate | se | CI95 |
| Intercept | 5.02 | 0.56 | 3.92 ; 6.12 |  | 5.04 | 0.54 | 3.97 ; 6.10 |  | 5.00 | 0.56 | 3.89 ; 6.09 |
| *min*(T-k,0) | 0.07 | 0.08 | -0.08 ; 0.22 |  | 0.05 | 0.06 | -0.06 ; 0.16 |  | 0.08 | 0.08 | -0.08 ; 0.24 |
| *max*(T-k,0) | 0.02 | 0.14 | -0.25 ; 0.30 |  | 0.01 | 0.14 | -0.26 ; 0.28 |  | 0.05 | 0.21 | -0.36 ; 0.45 |
| IT:24h | 1.16 | 0.47 | 0.24 ; 2.08 |  | 1.16 | 0.47 | 0.24 ; 2.08 |  | 1.16 | 0.47 | 0.24 ; 2.07 |
| IT:48h | 6.32 | 0.92 | 4.52 ; 8.11 |  | 6.33 | 0.92 | 4.53 ; 8.12 |  | 6.31 | 0.92 | 4.51 ; 8.10 |
| *min*(T-k,0) × CC | 0.01 | 0.08 | -0.15 ; 0.17 |  | 0.01 | 0.06 | -0.11 ; 0.13 |  | 0.01 | 0.09 | -0.16 ; 0.18 |
| *min*(T-k,0) × GG | 0.07 | 0.09 | -0.10 ; 0.24 |  | 0.06 | 0.06 | -0.07 ; 0.18 |  | 0.08 | 0.09 | -0.10 ; 0.26 |
| *max*(T-k,0) × CC | −0.07 | 0.15 | -0.37 ; 0.23 |  | -0.08 | 0.15 | -0.37 ; 0.22 |  | -0.11 | 0.22 | -0.54 ; 0.32 |
| *max*(T-k,0) × GG | 0.23 | 0.16 | -0.08 ; 0.53 |  | 0.26 | 0.15 | -0.04 ; 0.56 |  | 0.33 | 0.22 | -0.10 ; 0.77 |

^*^Threshold: temperature/THI value above which there is a significant increase in the DFI; DIC: Deviance Information Criterion; se: standard error; CI95: 95% confident intervals.
