# Supplementary material for: Influence of the Temperature and the Genotype of the HSP90AA1 Gene over Sperm Chromatin Stability in Manchega Rams
Source: PLoS One. 2014 Jan 21;9(1):e86107. doi: 10.1371/journal.pone.0086107 (PMC3897619; doi:10.1371/journal.pone.0086107)
Supplement: Table S2 — Summary of mixed model effects relating DFI values with Tave, Tmax and THI for the days 29 to 35 prior to semen collection.* (DOC) [file pone.0086107.s003.doc]

**Table S2.** Summary of mixed model effects relating DFI values with Tave, Tmax and THI for the days 29 to 35 prior to semen collection.^*^

| Parameter | xDFI |  |  |  |  |  |  |  |  |  |  |
| --- | --- | --- | --- | --- | --- | --- | --- | --- | --- | --- | --- |
| Variable | Tave |  |  |  | Tmax |  |  |  | THI |  |  |
|  | Threshold | 23.5˚C | DIC = 4385 |  | Threshold | 31.7˚C | DIC = 4866 |  | Threshold | 22.2 | DIC = 4279 |
|  | Estimate | se | CI95 |  | Estimate | se | CI95 |  | Estimate | se | CI95 |
| Intercept | 20.38 | 0.10 | 20.19 ; 20.58 |  | 19.14 | 0.14 | 18.87 ; 19.41 |  | 21.28 | 0.08 | 21.12 ; 21.45 |
| *min*(T-k,0) | 0.00 | 0.01 | -0.02 ; 0.03 |  | -0.10 | 0.02 | -0.13 ; -0.06 |  | 0.07 | 0.01 | 0.05 ; 0.09 |
| *max*(T-k,0) | 0.68 | 0.03 | 0.62 ; 0.73 |  | 0.53 | 0.02 | 0.48 ; 0.57 |  | 1.29 | 0.05 | 1.18 ; 1.40 |
| IT:24h | -0.03 | 0.10 | -0.22 ; 0.16 |  | -0.03 | 0.12 | -0.27 ; 0.20 |  | -0.04 | 0.09 | -0.22 ; 0.15 |
| IT:48h | 1.02 | 0.13 | 0.77 ; 1.28 |  | 0.85 | 0.14 | 0.57 ; 1.12 |  | 0.99 | 0.13 | 0.74 ; 1.24 |
| *min*(T-k,0) × CC | 0.02 | 0.02 | -0.01 ; 0.05 |  | 0.02 | 0.02 | -0.02 ; 0.06 |  | 0.02 | 0.01 | -0.01 ; 0.05 |
| *min*(T-k,0) × GG | 0.01 | 0.02 | -0.02 ; 0.04 |  | 0.01 | 0.02 | -0.03 ; 0.05 |  | 0.02 | 0.01 | -0.01 ; 0.04 |
| *max*(T-k,0) × CC | 0.02 | 0.03 | -0.04 ; 0.08 |  | 0.01 | 0.02 | -0.03 ; 0.05 |  | 0.02 | 0.07 | -0.11 ; 0.16 |
| *max*(T-k,0) × GG | 0.09 | 0.03 | 0.02 ; 0.15 |  | 0.04 | 0.02 | 0.00 ; 0.08 |  | 0.16 | 0.07 | 0.03 ; 0.30 |
|  |  |  |  |  |  |  |  |  |  |  |  |
| Parameter | tDFI |  |  |  |  |  |  |  |  |  |  |
| Variable | Tave |  |  |  | Tmax |  |  |  | THI |  |  |
|  | Threshold | 24.3˚C | DIC = 8240 |  | Threshold | 32.5˚C | DIC = 8114 |  | Threshold | 22.7 | DIC = 8243 |
|  | Estimate | se | CI95 |  | Estimate | se | CI95 |  | Estimate | se | CI95 |
| Intercept | 4.35 | 0.43 | 3.51 ; 5.19 |  | 3.68 | 0.50 | 2.70 ; 4.67 |  | 4.35 | 0.42 | 3.52 ; 5.18 |
| *min*(T-k,0) | 0.01 | 0.05 | -0.08 ; 0.11 |  | -0.22 | 0.06 | -0.34 ; -0.10 |  | 0.02 | 0.05 | -0.09 ; 0.12 |
| *max*(T-k,0) | 0.64 | 0.23 | 0.18 ; 1.10 |  | 0.49 | 0.08 | 0.33 ; 0.64 |  | 0.87 | 0.32 | 0.25 ; 1.50 |
| IT:24h | 1.16 | 0.46 | 0.26 ; 2.06 |  | 1.51 | 0.44 | 0.64 ; 2.38 |  | 1.16 | 0.46 | 0.26 ; 2.06 |
| IT:48h | 6.35 | 0.92 | 4.55 ; 8.14 |  | 6.76 | 0.80 | 5.19 ; 8.33 |  | 6.34 | 0.92 | 4.54 ; 8.14 |
| *min*(T-k,0) × CC | 0.01 | 0.06 | -0.10 ; 0.12 |  | -0.03 | 0.08 | -0.17 ; 0.11 |  | 0.01 | 0.07 | -0.12 ; 0.14 |
| *min*(T-k,0) × GG | 0.05 | 0.06 | -0.07 ; 0.16 |  | 0.04 | 0.08 | -0.11 ; 0.29 |  | 0.05 | 0.07 | -0.08 ; 0.19 |
| *max*(T-k,0) × CC | -0.16 | 0.31 | -0.76 ; 0.44 |  | 0.01 | 0.08 | -0.15 ; 0.17 |  | -0.22 | 0.42 | -1.04 ; 0.59 |
| *max*(T-k,0) × GG | 0.79 | 0.31 | 0.18 ; 1.39 |  | 0.16 | 0.08 | 0.00 ; 0.32 |  | 1.07 | 0.42 | 0.25 ; 1.90 |

^*^Threshold: temperature/THI value above which there is a significant increase in the DFI; DIC: Deviance Information Criterion; se: standard error; CI95: 95% confident intervals.
