# Supplementary material for: Influence of the Temperature and the Genotype of the HSP90AA1 Gene over Sperm Chromatin Stability in Manchega Rams
Source: PLoS One. 2014 Jan 21;9(1):e86107. doi: 10.1371/journal.pone.0086107 (PMC3897619; doi:10.1371/journal.pone.0086107)
Supplement: Table S3 — Summary of mixed model effects relating DFI values with Tave, Tmax and THI for the days 37 to 42 prior to semen collection.* (DOC) [file pone.0086107.s004.doc]

**Table S3.** Summary of mixed model effects relating DFI values with Tave, Tmax and THI for the days 37 to 42 prior to semen collection.^*^

| Parameter | xDFI |  |  |  |  |  |  |  |  |  |  |
| --- | --- | --- | --- | --- | --- | --- | --- | --- | --- | --- | --- |
| Variable | Tave |  |  |  | Tmax |  |  |  | THI |  |  |
|  | Threshold | 20.8˚C | DIC = 5112 |  | Threshold | 28.4˚C | DIC = 5187 |  | Threshold | 21.9 | DIC = 5102 |
|  | Estimate | se | CI95 |  | Estimate | se | CI95 |  | Estimate | se | CI95 |
| Intercept | 21.08 | 0.14 | 20.80 ; 21.36 |  | 21.31 | 0.15 | 21.03 ; 21.60 |  | 21.58 | 0.18 | 21.36 ; 21.93 |
| *min*(T-k,0) | 0.11 | 0.03 | 0.06 ; 0.16 |  | 0.09 | 0.02 | 0.06 ; 0.13 |  | 0.13 | 0.02 | 0.08 ; 0.17 |
| *max*(T-k,0) | 0.30 | 0.03 | 0.24 ; 0.35 |  | 0.25 | 0.03 | 0.19 ; 0.31 |  | 0.92 | 0.13 | 0.67 ; 1.18 |
| IT:24h | -0.03 | 0.13 | -0.29 ; 0.23 |  | -0.03 | 0.14 | -0.30 ; 0.24 |  | -0.03 | 0.13 | -0.29 ; 0.23 |
| IT:48h | 0.91 | 0.15 | 0.61 ; 1.21 |  | 0.95 | 0.16 | 0.65 ; 1.25 |  | 0.91 | 0.15 | 0.62 ; 1.20 |
| *min*(T-k,0) × CC | 0.02 | 0.03 | -0.04 ; 0.08 |  | 0.01 | 0.02 | -0.03 ; 0.06 |  | 0.01 | 0.02 | -0.03 ; 0.06 |
| *min*(T-k,0) × GG | 0.01 | 0.03 | -0.05 ; 0.07 |  | 0.00 | 0.02 | -0.04 ; 0.05 |  | 0.02 | 0.02 | -0.03 ; 0.07 |
| *max*(T-k,0) × CC | 0.01 | 0.03 | -0.05 ; 0.07 |  | 0.01 | 0.03 | -0.05 ; 0.07 |  | 0.04 | 0.12 | -0.20 ; 0.28 |
| *max*(T-k,0) × GG | 0.05 | 0.03 | -0.00 ; 0.11 |  | 0.06 | 0.03 | -0.01 ; 0.12 |  | 0.24 | 0.12 | -0.00 ; 0.48 |
|  |  |  |  |  |  |  |  |  |  |  |  |
| Parameter | tDFI |  |  |  |  |  |  |  |  |  |  |
| Variable | Tave |  |  |  | Tmax |  |  |  | THI |  |  |
|  | Threshold | 22.38˚C | DIC = 8252 |  | Threshold | 28.2˚C | DIC = 8250 |  | Threshold | 21.9 | DIC = 8253 |
|  | Estimate | se | CI95 |  | Estimate | se | CI95 |  | Estimate | se | CI95 |
| Intercept | 3.03 | 0.62 | 1.80 ; 4.24 |  | 3.28 | 0.49 | 2.33 ; 4.23 |  | 3.06 | 0.61 | 1.86 ; 4.27 |
| *min*(T-k,0) | -0.07 | 0.08 | -0.22 ; 0.07 |  | -0.04 | 0.06 | -0.17 ; -0.08 |  | -0.07 | 0.08 | -0.23 ; 0.08 |
| *max*(T-k,0) | 0.99 | 0.23 | 0.54 ; 1.44 |  | 0.45 | 0.10 | 0.25 ; 0.64 |  | 1.98 | 0.46 | 1.08 ; 2.88 |
| IT:24h | 1.17 | 0.46 | 0.27 ; 2.08 |  | 1.17 | 0.46 | 0.27 ; 2.07 |  | 1.18 | 0.46 | 0.27 ; 2.08 |
| IT:48h | 6.37 | 0.92 | 4.56 ; 8.17 |  | 6.36 | 0.92 | 4.55 ; 8.16 |  | 6.36 | 0.92 | 4.55 ; 8.17 |
| *min*(T-k,0) × CC | 0.00 | 0.08 | -0.15 ; 0.15 |  | -0.01 | 0.08 | -0.16 ; 0.14 |  | 0.00 | 0.08 | -0.16 ; 0.15 |
| *min*(T-k,0) × GG | 0.04 | 0.08 | -0.12 ; 0.20 |  | 0.02 | 0.08 | -0.14 ; 0.18 |  | 0.04 | 0.08 | -0.12 ; 0.21 |
| *max*(T-k,0) × CC | -0.12 | 0.23 | -0.56 ; 0.33 |  | -0.06 | 0.11 | -0.28 ; 0.16 |  | -0.23 | 0.46 | -1.13 ; 0.67 |
| *max*(T-k,0) × GG | 0.03 | 0.23 | -0.43 ; 0.49 |  | 0.01 | 0.11 | -0.21 ; 0.24 |  | 0.13 | 0.47 | -0.79 ; 1.05 |

^*^Threshold: temperature/THI value above which there is a significant increase in the DFI; DIC: Deviance Information Criterion; se: standard error; CI95: 95% confident intervals.
