# Supplementary material for: Influence of the Temperature and the Genotype of the HSP90AA1 Gene over Sperm Chromatin Stability in Manchega Rams
Source: PLoS One. 2014 Jan 21;9(1):e86107. doi: 10.1371/journal.pone.0086107 (PMC3897619; doi:10.1371/journal.pone.0086107)
Supplement: Table S4 — Summary of mixed model effects relating DFI values with Tave, Tmax and THI for the days 45 to 47 prior to semen collection.* (DOC) [file pone.0086107.s005.doc]

**Table S4.** Summary of mixed model effects relating DFI values with Tave, Tmax and THI for the days 45 to 47 prior to semen collection.^*^

| Parameter | xDFI |  |  |  |  |  |  |  |  |  |  |
| --- | --- | --- | --- | --- | --- | --- | --- | --- | --- | --- | --- |
| Variable | Tave |  |  |  | Tmax |  |  |  | THI |  |  |
|  | Threshold | 23.2˚C | DIC = 4656 |  | Threshold | 31.3˚C | DIC = 4508 |  | Threshold | 22.2 | DIC = 4682 |
|  | Estimate | se | CI95 |  | Estimate | se | CI95 |  | Estimate | se | CI95 |
| Intercept | 22.35 | 0.11 | 22.13 ; 22.57 |  | 21.88 | 0.11 | 21.67 ; 22.09 |  | 22.42 | 0.11 | 22.20 ; 22.64 |
| *min*(T-k,0) | 0.14 | 0.01 | 0.12 ; 0.16 |  | 0.09 | 0.02 | 0.07 ; 0.11 |  | 0.16 | 0.01 | 0.14 ; 0.19 |
| *max*(T-k,0) | 0.53 | 0.04 | 0.45 ; 0.62 |  | 0.57 | 0.03 | 0.51 ; 0.64 |  | 0.77 | 0.06 | 0.64 ; 0.89 |
| IT:24h | -0.02 | 0.11 | -0.24 ; 0.19 |  | -0.03 | 0.10 | -0.23 ; 0.17 |  | -0.02 | 0.11 | -0.24 ; 0.19 |
| IT:48h | 0.96 | 0.13 | 0.70 ; 1.21 |  | 1.01 | 0.13 | 0.76 ; 1.26 |  | 0.95 | 0.13 | 0.69 ; 1.20 |
| *min*(T-k,0) × CC | 0.01 | 0.01 | -0.02 ; 0.03 |  | 0.01 | 0.01 | -0.01 ; 0.03 |  | 0.01 | 0.01 | -0.02 ; 0.04 |
| *min*(T-k,0) × GG | 0.02 | 0.01 | -0.00 ; 0.05 |  | 0.02 | 0.01 | -0.00 ; 0.04 |  | 0.03 | 0.01 | -0.01 ; 0.05 |
| *max*(T-k,0) × CC | 0.01 | 0.05 | -0.09 ; 0.12 |  | 0.01 | 0.04 | -0.07 ; 0.10 |  | 0.02 | 0.08 | -0.14 ; 0.18 |
| *max*(T-k,0) × GG | 0.06 | 0.05 | -0.05 ; 0.17 |  | 0.06 | 0.04 | -0.02 ; 0.15 |  | 0.09 | 0.08 | 0.08 ; 0.25 |
|  |  |  |  |  |  |  |  |  |  |  |  |
| Parameter | tDFI |  |  |  |  |  |  |  |  |  |  |
| Variable | Tave |  |  |  | Tmax |  |  |  | THI |  |  |
|  | Threshold | 19.2˚C | DIC = 8239 |  | Threshold | 26.00˚C | DIC = 8114 |  | Threshold | 18.6 | DIC = 8240 |
|  | Estimate | se | CI95 |  | Estimate | se | CI95 |  | Estimate | se | CI95 |
| Intercept | 4.55 | 0.51 | 3.55 ; 5.54 |  | 3.68 | 0.50 | 2.70 ; 4.67 |  | 4.44 | 0.52 | 3.42 ; 5.45 |
| *min*(T-k,0) | 0.08 | 0.07 | -0.05 ; 0.22 |  | -0.22 | 0.06 | -0.34 ; -0.10 |  | 0.08 | 0.08 | -0.07 ; 0.23 |
| *max*(T-k,0) | 0.29 | 0.10 | 0.09 ; 0.48 |  | 0.49 | 0.08 | 0.33 ; 0.64 |  | 0.39 | 0.13 | 0.13 ; 0.64 |
| IT:24h | 1.18 | 0.46 | 0.28 ; 2.08 |  | 1.51 | 0.44 | 0.64 ; 2.38 |  | 1.19 | 0.46 | 0.28 ; 2.08 |
| IT:48h | 6.20 | 0.92 | 5.15 ; 7.99 |  | 6.76 | 0.80 | 5.19 ; 8.33 |  | 6.20 | 0.92 | 4.40 ; 8.01 |
| *min*(T-k,0) × CC | 0.01 | 0.08 | -0.14 ; 0.15 |  | -0.03 | 0.08 | -0.17 ; 0.11 |  | 0.01 | 0.08 | -0.16 ; 0.17 |
| *min*(T-k,0) × GG | 0.06 | 0.08 | -0.09 ; 0.22 |  | 0.04 | 0.08 | -0.11 ; 0.29 |  | 0.07 | 0.09 | -0.10 ; 0.24 |
| *max*(T-k,0) × CC | -0.03 | 0.12 | -0.26 ; 0.21 |  | 0.01 | 0.08 | -0.15 ; 0.17 |  | -0.04 | 0.15 | -0.34 ; 0.26 |
| *max*(T-k,0) × GG | 0.10 | 0.12 | -0.14 ; 0.33 |  | 0.16 | 0.08 | 0.00 ; 0.32 |  | 0.12 | 0.15 | -0.18 ; 0.42 |

^*^Threshold: temperature/THI value above which there is a significant increase in the DFI; DIC: Deviance Information Criterion; se: standard error; CI95: 95% confident intervals.
